# Adverse Outcomes of IVF/ICSI Pregnancies Vary Depending on Aetiology of Infertility

**DOI:** 10.5402/2012/451915

**Published:** 2012-04-09

**Authors:** Paula Kuivasaari-Pirinen, Kaisa Raatikainen, Maritta Hippeläinen, Seppo Heinonen

**Affiliations:** ^1^Department of Obstetrics and Gynaecology, Kuopio University Hospital, 70211 Kuopio, Finland; ^2^Faculty of Health Sciences, University of Eastern Finland, 70211 Kuopio, Finland

## Abstract

*In vitro* fertilization (IVF) is a risk factor for pregnancy, but there have been few studies on the effect of infertility's aetiology. Thus, we have assessed the role of aetiology on IVF pregnancy outcomes in a retrospective cohort study comparing the outcomes of IVF singleton pregnancies with those of spontaneous pregnancies in the general Finnish population. The study group consisted of 255 women with births resulting from singleton IVF pregnancies. Six subgroups were formed according to the following causes of infertility: anovulation (27%), endometriosis (19%), male factor (17%), tubal factor (15%), polycystic ovary syndrome (11%), and unexplained infertility (12%). The reference group consisted of 26,870 naturally conceived women. Adjusted odds ratios (AORs), for confounding factors such as age and parity, were estimated using logistic regression analysis. Women with endometriosis and anovulation had increased risks of preterm birth (AOR 3.25, 95% CI 1.5–7.1 and AOR 2.1, and 95% CI 1.0–4.2, resp.), while women in couples with male factor infertility had a twofold risk of admission to neonatal intensive care (AOR 2.5, 95% CI 1.2–5.3). The findings show that the aetiology of infertility influenced the obstetrics outcome, and that pooling results may obscure some increased risks among subgroups.

## 1. Introduction

Since the first child was born after in vitro fertilization (IVF) [1], more than 30 years ago, IVF and other assisted reproductive technologies (ARTs) have developed rapidly, and the accessibility to treatment has improved. The obvious goals of all such treatments are to achieve a pregnancy and subsequent birth of a healthy infant, but overall IVF pregnancies are often seen as risky [2, 3], mainly due to multiple gestations [4–6]. However, even singleton ART pregnancies are associated with increased incidences of preterm birth, low birth weight, small-for-gestational-age (SGA) infants, and obstetric complications such as preeclampsia, placental abruption, and placenta praevia [2, 7, 8]. The increased risks can be partly explained by maternal characteristics such as increased age [9] and underlying infertility [10], but the effects of the infertility's etiology as a potential etiologic factor of obstetric complications and adverse neonatal outcomes have been sparsely investigated [11]. Thus, the aim of the presented study was to elucidate associations between indications for ART with the incidence of adverse obstetric outcomes among ART singleton pregnancies.

## 2. Materials and Methods

In this cohort study concerning the outcomes of ART, including IVF, intracytoplasmic sperm injection (ICSI), and frozen-thawed cycles, first singleton ART pregnancies of 255 women were compared with spontaneous singleton pregnancies in the general population during 1996–2007 at the University Hospital of Kuopio, Finland. In both groups, considered pregnancies were restricted to those that proceeded to delivery (≥22 weeks of gestation or birth weight of at least 500 g). In addition, pregnancies with major foetal malformations were excluded, since they carry higher risks of obstetric and neonatal complications. The study group was divided into six subgroups, based on the indication for ART: anovulation 27% (*n* = 68), endometriosis 19% (*n* = 49), male factor infertility 17% (*n* = 43), polycystic ovary syndrome (PCOS) 11% (*n* = 27), tubal factor 15% (*n* = 38), and unexplained infertility 12% (*n* = 30). The aetiology in each case was diagnosed by laparoscopy, ultrasonography, and laboratory parameters (when appropriate), and the sperm of every couple was analyzed. Unexplained infertility was defined as infertility lasting at least one year for which no explanatory factor was identified. Preconditions for ART treatment among women suffering from anovulation, PCOS, and unexplained infertility were unsuccessful ovulation induction and three inseminations. The reference group comprised 26.870 spontaneous singleton pregnancies. The pregnancies and deliveries of the study and reference groups were handled in the same tertiary hospital, and ART treatments were carried out in the same unit. The data were collected from Kuopio University Hospital birth register and databases of the Obstetrics and Fertility Outpatient Departments.

Kuopio University Hospital birth register contains information systematically collected from a questionnaire completed by every woman who delivered at Kuopio University Hospital, with questions concerning maternal characteristics such as marital status, smoking habits, chronic illnesses, and previous pregnancies. In addition, the data were supplemented by information from the women's case notes which they carry with them, notes made by nurses or doctors during visits to the maternity clinic, and information recorded by the midwife who took care of the birth concerning the birth and neonatal period up to the age of one week.

A standard long IVF protocol with gonadotropin-releasing hormone (GnRH) agonists and gonadotrophins was used as previously described [12]. In addition to cases of male factor infertility, ICSI was used according to our practice in all cases of unexplained infertility when a woman had ovulated after the use of either clomifene or gonadotrophins but failed to become pregnant. If the number of oocytes retrieved was >10, ICSI was used in half of them.

### 2.1. Definitions

Deliveries at <37 weeks of gestation were defined as premature and low birth weight (LBW) as <2500 g. Infants were classified as SGA when their birth weight was below the 10th percentile adjusted for our population [13]. Preeclampsia was diagnosed if proteinuria exceeded 0.5 g/day and blood pressure exceeded 149/90 mm Hg repeatedly, and gestational diabetes if a single abnormal value in oral glucose tolerance tests was recorded.

The study was approved by the ethics committee and the institutional review board of our institution. Statistical analyses were carried out with SPSS version 16.0 software (SPSS Inc., Chicago, IL, USA). Statistically significant differences were determined using the Mantel-Haenszel *χ* ^2^ test, Fisher's exact test when *n* was <5, or Student's *t*-test, as appropriate. A value of *P* < 0.05 was considered statistically significant. Multivariate analysis of significant or nearly significant (*P* < 0.1) correlations between maternal characteristics (such as age over 35 years, primiparity, marital status, chronic illnesses, and smoking during pregnancy) and the incidence of obstetric outcomes was based on multiple logistic regression analysis (SAS, Institute Inc., Cary, NC, USA, version 9.1). Odds ratios (ORs) with 95% confidence intervals (CIs) were calculated for each subgroup separately and for the pooled IVF/ICSI group before and after adjustment for maternal characteristics. Obstetric outcomes in the subgroups were compared with those in the reference group.

## 3. Results

### 3.1. Maternal Characteristics

The mothers in the ART group were older, smoked less, and were more often nulliparous and married than the mothers in the reference group. More detailed information is provided in Table 1.

### 3.2. Pregnancy Complications

The mothers in the ART group suffered more preeclampsia than those in the reference group, this being a particular problem of the women with tubal factor infertility. Furthermore, the pooled study group showed a greater incidence of placenta praevia than the reference group, and in the subgroups, this was particularly the case among mothers with endometriosis and those in the male factor infertility group. Placental abruption was found to be accumulated in women with unexplained infertility. Table 1 shows detailed information.

### 3.3. Neonatal Outcome

Table 2 summarizes the effects of the aetiology of infertility on neonatal outcome. After adjustment for confounding factors, preterm birth was found to be particularly a problem for women with endometriosis and those suffering from anovulation. The incidence of SGA infants was increased among women in the male factor infertility and unexplained infertility subgroups. The fewest complications appeared among the ART mothers who suffered from PCOS or tubal factor infertility.

## 4. Discussion

The results of the study show that the pooled group of women with singleton ART pregnancies had increased risks of preterm birth, LBW, and NICU admission, independently of age and parity, compared to spontaneously conceived women. These findings are in accordance with previous studies [2, 7], although contrasting results have also been presented [14]. However, the main finding of the present study was that the ART subgroups did not show a uniform pattern of adverse outcomes. The rate of preterm deliveries was fairly constantly two- to threefold higher across all except the unexplained fertility aetiological subgroups, but SGA was mainly associated with couples suffering from male factor or unexplained infertility. The net effects of concomitant SGA and prematurity risks in the male factor subgroup in turn resulted in the highest NICU admission rate, whereas no such risk was observed for the unexplained infertility subgroup. These results imply that the obstetric risks associated with IVF should not be pooled, and the aetiology of infertility should be accounted for when its outcomes are considered.

In addition, the incidence of chronic illnesses was highest among women with infertility due to anovulation. These, and women with PCOS, had more than a twofold risk of preterm birth but no increased SGA risk after adjustment for confounding factors. The mean birth weight for these subgroups did not significantly differ from that of the reference group and was even somewhat higher than that of other ART pregnancies.

Women with endometriosis had the highest risk of preterm birth—in accordance with findings of other studies [15, 16]—and consequently their infants had the lowest mean birth weight, but interestingly their SGA risk was low, and thus their need for intensive neonatal care was comparable to that of the other ART subgroups. Women with tubal factor infertility had a very similar outcome pattern, although they experienced preeclampsia more often than the reference group.

The male factor and unexplained infertility subgroups both had a twofold increased risk of SGA, accompanied by increased prematurity risk in the former but not the latter. The NICU admission rate was subsequently highest for the male factor subgroup and lowest for the unexplained infertility subgroup. Women with unexplained infertility also had significantly more placental problems such as miscarriages and abruptions than the reference group and lowest mean birth weight, when the mean gestational age was taken into account. Similarly, Pandian et al. [17] found that a group of Scottish women with unexplained infertility had more preeclampsia, placental abruption, and preterm births than women in the general obstetric population. However, contrasting results have also been reported; according to another Finnish study, IVF pregnancies of women with unexplained infertility had similar outcomes to those of spontaneous pregnancies [18].

The present study was a pilot study with a relatively small sample size with inadequate power to analyse differences between the subgroups, which would have been very interesting. Nevertheless, one of the strengths was the extensive database including maternal characteristics and information on the course of pregnancy and delivery collected prospectively enabling wide adjustments for confounding factors.

In conclusion, the aetiology of infertility affected obstetric complications and neonatal outcomes. The risk of adverse outcome among ART pregnancies varied significantly depending on the cause of infertility. All except the unexplained infertility IVF subgroups had a two- to threefold elevated risk of preterm birth, and the infants of women with male factor infertility had the highest (ca. twofold) risk of admission to NICU in comparison with the general obstetric population. The widest variation was in the occurrence of SGA infants. If only pooled results had been analysed, the overall SGA risk would have remained unnoticed. According to our results, risks of SGA were elevated for women with male factor and unexplained infertility, while women with PCOS and those with tubal factor infertility had the fewest obstetric complications and the best neonatal outcomes.

Our results clearly show that women undergoing ART are a heterogeneous group, and this should be taken into consideration both when planning IVF/ICSI treatment and during antenatal care. Further studies are needed to clarify the causes of the wide observed variation in adverse pregnancy outcomes.

## Figures and Tables

**Table 1:** Maternal, treatment, and pregnancy characteristics and neonatal outcome in the study groups compared with the reference groups.

			Maternal characteristics					
	Ref. group 26 870	ART 255	Anovul. 68	Endometriosis 49	Male factor 43	Tubal factor 38	PCOS 27	Unexplain 30
Age >35 years %	13.6	26.7	29.4	24.5	18.6	42.1	11.1	30.0
*P* value		<0.0001	<0.001	<0.05	NS	<0.0001	NS	NS
BMI^a^ >25%	24.0	25.5	28.4	20.4	22.0	23.7	48.2	13.8
*P* value		NS	NS	NS	NS	NS	<0.01	NS
Married %	55.3	72.2	70.6	77.6	74.4	57.9	77.8	76.7
*P* value		<0.0001	<0.05	<0.01	<0.05	NS	NS	<0.05
Nulliparous %	40.1	75.7	79.4	83.7	72.1	63.2	63.0	86.7
*P* value		<0.0001	<0.0001	<0.0001	<0.0001	<0.005	<0.05	<0.0001
Smoking^b^ %	20.6	11.2	9.1	8.2	12.2	18.9	11.5	10.0
*P* value		<0.001	<0.05	<0.05	NS	NS	NS	NS
Prev. miscarriage %	19.1	16.1	13.2	16.3	14.0	15.8	18.5	23.3
*P* value		NS	NS	NS	NS	NS	NS	NS
Prev. foetal demise %	1.2	1.2	4.4	0	0	0	0	0
*P* value		NS	<0.05	NS	NS	NS	NS	NS
Chronic illnesses %	11.3	12.2	26.5	10.2	7.0	5.3	7.4	3.3
*P* value		NS	<0.0001	NS	NS	NS	NS	NS

			Treatment characteristics					

Dur. of infertility (y)		3.1	3.2	3.2	2.9	3.1	3.1	3.2
N : o of ovarian stimul		1.8	2.1	1.8	1.8	1.6	1.5	1.6
IVF pregnancy %		41.2	41.2	51.0	0	65.8	51.9	30.0
ICSI pregnancy %		45.9	48.5	24.5	88.4	26.3	25.9	56.7
Frozen-thawed preg %		14.5	10.3	24.5	11.6	7.9	22.2	13.3

				Pregnancy characteristics				

Gestational diabetes %	10.6	11.9	11.8	14.3	4.7	13.2	22.2	6.7
*P* value		NS	NS	NS	NS	NS	NS	NS
Pre-eclampsia %	3.6	6.3	4.4	4.1	7.0	10.5	7.4	6.7
*P* value		<0.05	NS	NS	NS	<0.05	NS	NS
Placenta praevia %	0.6	3.6	1.5	6.1	4.7	2.6	3.7	3.3
*P* value		<0.0001	NS	<0.005	<0.05	NS	NS	NS
Placental abruption %	0.6	1.2	0	0	2.3	0	0	6.7
*P* value		NS	NS	NS	NS	NS	NS	<0.05

				Neonatal outcome				

Gestational age, d ± SD	277 ± 15	273 ± 18	274 ± 17	268 ± 23	274 ± 12	275 ± 21	276 ± 13	274 ± 20
*P* value		<0.005	<0.05	<0.05	NS	NS	NS	NS
Birthweight, g ± SD	3488 ± 606	3332 ± 688	3365 ± 683	3185 ± 697	3301 ± 587	3413 ± 821	3591 ± 639	3208 ± 641
*P* value		<0.005	NS	<0.005	<0.05	NS	NS	<0.01

^a^Body mass index (BMI), kg/m^2^, ^b^smoking during pregnancy >5 cigarettes/day.

**Table 2:** Neonatal outcomes in the study groups compared with the reference group.

Outcome *n*	Ref. group 26 870	ART 255	Anovulation 68	Endometriosis 49	Male factor 43	Tubal factor 38	PCOS 27	Unexplained 30
*Preterm birth *								
%	6.3	12.7	13.4	18.6	9.8	10.5	14.8	6.7
*P*-value		<0.0001	<0.05	<0.005	NS	NS	NS	NS
OR		**2.17**	**2.33**	**3.43**	1.62	1.77	2.61	1.07
95% CI		1.48–3.17	1.15–4.71	1.59–7.42	0.58–4.53	0.63–4.99	0.90–7.57	0.26–4.51
AOR		**2.12**	**2.07**	**3.25**	1.80	1.71	2.85	1.06
95% CI		1.44–3.11	1.01–4.23	1.50–7.07	0.64–5.11	0.60–4.85	0.98–8.30	0.25–4.48

*LBW *								
%	4.7	8.7	7.4	10.2	9.3	10.5	3.7	10.0
*P*-value		<0.005	NS	NS	NS	NS	NS	NS
OR		**1.93**	1.62	2.32	2.09	2.40	0.79	2.27
95% CI		1.24–3.00	0.65–4.04	0.92–5.86	0.75–5.87	0.85–6.79	0.11–5.79	0.69–7.49
AOR		**1.83**	1.35	2.13	2.27	2.33	0.87	2.18
95% CI		1.17–2.86	0.54–3.40	0.84–5.41	0.80–6.42	0.82–6.63	0.12–6.49	0.65–7.24

*SGA *								
%	9.7	12.4	11.8	6.3	19.1	7.9	7.4	23.3
*P*-value		NS	NS	NS	NS	NS	NS	<0.05
OR		1.30	1.24	0.62	**2.18**	0.80	0.74	**2.83**
95% CI		0.90–1.90	0.59–2.59	0.19–1.20	1.01–4.72	0.24–2.59	0.18–3.14	1.21–6.59
AOR		1.08	1.02	0.49	1.78	0.69	0.78	2.29
95% CI		0.73–1.59	0.49–2.16	0.15–1.59	0.78–4.06	0.21–2.25	0.18–3.33	0.97–5.41

*NICU admiss*								
%	9.5	16.6	14.7	18.4	20.9	15.8	18.5	10.0
*P*-value		<0.0005	NS	<0.05	<0.05	NS	NS	NS
OR		**1.87**	1.64	**2.14**	**2.51**	1.78	2.16	1.05
95% CI		1.34–2.60	0.84–3.21	1.04–4.01	1.20–5.24	0.74–4.26	0.82–5.70	0.32–3.48
AOR		**1.64**	1.32	1.78	**2.49**	1.62	1.98	0.92
95% CI		1.17–2.30	0.67–2.60	0.86–3.68	1.18–5.26	0.67–3.90	0.74–5.28	0.28–3.05

Preterm birth <37 gestational weeks, LBW: low birth weight (<2500 g), SGA: small for gestational age (fetal indexes < the 10th percentile adjusted for Finnish population), NICU: neonatal intensive care unit.

AOR: OR adjusted for age, parity, BMI, smoking, previous foetal deaths and miscarriages, chronic illness, and marital status.
